# Hsp70 Is a Potential Therapeutic Target for Echovirus 9 Infection

**DOI:** 10.3389/fmolb.2020.00146

**Published:** 2020-07-17

**Authors:** Yang Wang, Hui Zhang, Dongbo Ma, Xiang Deng, Dongdong Wu, Fang Li, Qiuge Wu, Hong Liu, Jing Wang

**Affiliations:** Department of Respiratory and Critical Care Medicine, The First Affiliated Hospital of Zhengzhou University, Zhengzhou, China

**Keywords:** echovirus, proteomics, encephalitis, RT-PCR, Hsp70, differential protein expression

## Abstract

Echovirus is an important cause of viral pneumonia and encephalitis in infants, neonates, and young children worldwide. However, the exact mechanism of its pathogenesis is still not well understood. Here, we established an echovirus type 9 infection mice model, and performed two-dimensional gel electrophoresis (2DE) and tandem mass spectrometry (MS/MS)-based comparative proteomics analysis to investigate the differentially expressed host proteins in mice brain. A total of 21 differentially expressed proteins were identified by MS/MS. The annotation of the differentially expressed proteins by function using the UniProt and GO databases identified one viral protein (5%), seven cytoskeletal proteins (33%), six macromolecular biosynthesis and metabolism proteins (28%), two stress response and chaperone binding proteins (9%), and five other cellular proteins (25%). The subcellular locations of these proteins were mainly found in the cytoskeleton, cytoplasm, nucleus, mitochondria, and Golgi apparatus. The protein expression profiles and the results of quantitative RT-PCR in the detection of gene transcripts were found to complement each other. The differential protein interaction network was predicted using the STRING database. Of the identified proteins, heat shock protein 70 (Hsp70), showing consistent results in the proteomics and transcriptomic analyses, was analyzed through Western blotting to verify the reliability of differential protein expression data in this study. Further, evaluation of the function of Hsp70 using siRNA and quercetin, an inhibitor of Hsp70, showed that Hsp70 was necessary for the infection of echovirus type 9. This study revealed that echovirus infection could cause the differential expression of a series of host proteins, which is helpful to reveal the pathogenesis of viral infection and identify therapeutic drug targets. Additionally, our results suggest that Hsp70 could be a useful therapeutic host protein target for echovirus infection.

## Introduction

Echoviruses are one of the most common causative agents for aseptic meningitis, as well as viral pneumonia, worldwide and are especially devastating for the newborn population. It can cause severe hepatitis, severe pneumonia, and neurological diseases, including meningitis and encephalitis, and even death (Morosky et al., 2019). The virus infects through the pharynx mucosa and proliferates into the blood. Viremia has also been reported in cases of viral pneumonia echovirus type 9 infection (Yoshioka and Horstmann, 1960; Aguado et al., 2014). Severe echovirus type 9 infections have been implicated in the acute development of type I diabetes mellitus (Vreugdenhil et al., 2000; Paananen et al., 2003; Cabrera-Rode et al., 2005). It has also been shown to induce age-dependent paralysis in infant mice (Bultmann et al., 1983). Although echoviruses comprise a large number of serotypes, studies on echovirus pathogenesis are relatively limited, especially for echovirus type 9. Herpes simplex virus and arboviruses are common causes of encephalitis; enteroviruses are more associated with mild central nervous system infections, such as aseptic meningitis, and one study has reported that the majority of encephalitis cases and severe cases of viral pneumonia were caused by echovirus type 9 (Dalwai et al., 2009).

Echovirus infections are prevalent and frequently asymptomatic. However, they can be severe and life-threatening depending on the virus types, its effects on host protein expression, and physiological metabolism. An earlier study suggested that the determinants of capsid proteins might cause the divergent behaviors of different virus strains and-possibly-influence pathogenicity (Rosenwirth and Eggers, 1982). In temperate regions, the disease occurs during summer and autumn, whereas, in the tropics, it can occur all year round. The clinical symptoms of echovirus infection are strongly correlated with age: central nervous system diseases are more common in patients aged 5–15 years; myocarditis is more prevalent in patients aged 20–40 years; severe infections, including myocarditis, central nervous system diseases, and sepsis occur in neonates and infants; and hand, foot, and mouth disease occurs in children younger than 5 years old (Muehlenbachs et al., 2015). By far, studies on the function of host factors in echovirus infection have been mostly limited to cell receptors or cytokine levels, while studies on other cellular proteins, especially for echovirus type 9, have been rare. Huttunen et al. (1997) found that VLA-2 integrin can be used as a cellular receptor for echovirus 1, and this ligand binding of integrin could ultimately trigger signal transduction pathways activating immediate-early genes. Ichiyama et al. (2005) demonstrated that the concentrations of the cerebrospinal fluid IFN-γ, IL-2, IL-6, and IL-10 were elevated in mumps meningitis, while the concentrations of the cerebrospinal fluid IFN-γ, IL-2, and IL-6 levels were elevated in meningitis. Moreover, the expression levels of the matrix metalloproteinase-9 and the tissue inhibitor of metalloproteinase-1 in the cerebrospinal fluid of patients with meningitis caused by echovirus type 30 were elevated (Sulik et al., 2008). In the limited host factor correlation studies of echovirus, it was proved that the complement regulator decay-accelerating factor (DAF, CD55) as a cellular receptor of echovirus type 12, and bind to a two-domain fragment of it (Bhella et al., 2004). In addition, heparan sulfate may also be an interaction factor at the cell surface for echoviruses (Goodfellow et al., 2001). In view of echovirus studies being limited to epidemiological studies and a small number of host factor studies, it is necessary to use proteomics to study the differential host proteins caused by the virus infection, which might play a key role in revealing the pathogenesis of echovirus and the discovery of therapeutic drug targets. In the present study, we have analyzed the expression profiles of different host proteins in mice brain using an echovirus type 9 infection mice model, and identified the differentially expressed protein Hsp70, as a possible therapeutic target for echovirus type 9 infections.

## Materials and Methods

### Animals, Cells, Chemicals, and Viruses

Kunming mice (SPF class) were purchased from the Center of Experimental Animal of the Zhengzhou University. The rhabdomyosarcoma (RD) cell line, derived from a human rhabdomyosarcoma, was purchased from ATCC and cultured in Dulbecco's modified Eagle medium (DMEM) (Corning, NY) with 4.5 g of glucose/L and 10% fetal calf serum. The cells were maintained in our lab. The echovirus type 9 strain was isolated from the cerebrospinal fluid of a meningitis patient. Quercetin and rabbit polyclonal antibodies (pAb) against GAPDH were purchased from Sigma Chemicals (St. Louis, MO) and Hangzhou GoodHere Biotechnology Co. Ltd (Hangzhou, China), respectively. Rabbit monoclonal [EPR16892] to Hsp70 antibody and Goat anti-Rabbit IgG H&L (HRP) were purchased from Abcam (Shanghai, China). HSP 70 siRNA (h) (sc-29352) and Control siRNAs (sc-37007) were purchased from Santa Cruz Biotechnology, Inc.

### Mice Challenge Experiment and Protein Preparation

Three days old Kunming Mice were randomly divided into two groups, and infected with 2–5 × 106 pfu of echovirus type 9 by intracranial injection, while the mock-infected group was treated with normal cell culture supernatant. The mice were sacrificed at 24, 48, and 96 h after the challenge, and their brains were extracted, and quickly ground in liquid nitrogen. An appropriate volume of protein lysis buffer was added after weighing the sample. The exact method for the preparation of samples was described previously by Wu et al. (2009). The concentrations of the samples were determined by the Bradford method. The study was approved in 2017 by the First Affiliated Hospital of Zhengzhou University (Study approval number: 2017050).

### Two-Dimensional Electrophoresis (2-DE) and Silver Staining

Two dimensional electrophoresis (2DE) of the brain proteins was performed using a previously published method (Wu et al., 2012; Zhang et al., 2013). Briefly, the commercial 24-cm long ReadyStrip IPG strips (pH 5–8, linear, Bio-Rad) in PROTEAN IEF (isoelectric focusing) Cell (Bio-Rad) were used for the first dimension separation. Each strip was loaded with 250 μg protein in total. After the IEF is completed, the strips were equilibrated with an IEF buffer containing 1% dithiothreitol (DTT) and 2.5% iodoacetamide for 15 min. Subsequently, the strips were run on the 11% SDS-PAGE gel at 80 V for 40 min, followed by 200 V until the second dimension separation finished. All gels were silver-dyed with an improved mass spectroscopy-compatible method and scanned at a resolution of 500 dpi on a Typhoon 9,410 Variable Mode Imager (Amersham Biosciences).

### Image Analysis of the Gels

Image analysis of the gels included the spot detection, spot matching, quantitative intensity analysis, and background substraction, which were carried out with the analysis software PDQuest 2-D 8.0.1 software according to the manufacturer's protocol. In order to avoid the difference in dyeing treatment of different gels, the total gray level of the whole gel was normalized and compared according to the ratio of the total gray level of each protein point at all protein points of the gel (Kholghi et al., 2019). Only those spots with a ratio >2.0 or <0.5 between the infected group and the control group were considered as differential proteins and were subjected to identification by MS/MS.

### Identification of the Protein Spots

The differentially expressed protein points were cut from the two-dimensional electrophoresis gels. Elution for another hour was performed with 100 μl of 25 mM ammonium bicarbonate containing 50% ACN. The proteins were identified by 4,700 MALDI-TOF/TOF Proteomics Analyzer (Applied Biosystems, Foster City, CA, USA) following the procedure described in a previous study (Zheng et al., 2008). In order to remove redundant proteins with different names and accession numbers from the database, only those belonging to *Mus musculus* or having the highest protein score (top rank) were selected from the multi-protein family.

### RNA Extraction and RT-qPCR

RNA in brain tissues of virus challenged and mock-infected mice were extracted with total RNA extraction kit for animal tissues (DP431, Tiangen Biotech Co., LTD, Beijing, China) according to the manufacturer's protocol. The concentrations of the extracted RNA were measured using a spectrophotometer (260/280 nm). Subsequently, 1 μg RNA of each sample was transcribed by HiScript® II Q RT SuperMix for qPCR (+gDNA wiper) (Vazyme, Nanjing, China) to obtain the cDNA. With reference to the gene sequences of the proteins identified by mass spectrometry, the specific primers (Table S1) for simultaneous detection of different target genes were designed using PrimerQuest Tool of the Integrated DNA Technologies (https://sg.idtdna.com/site/account/login?returnurl=%2FPrimerquest%2FHome%2FIndex). The RT-PCR was performed by using the 7,500 Real-Time System (Applied Biosystems) with ChamQ SYBR Color qPCR Master Mix (Vazyme, Nanjing, China). The quantitative analysis of data using the obtained standard curve was performed in the 7,500 System SDS software Version 1.4.1 following the relative quantification (ΔΔCt) model (Applied Biosystems). The mock-infected mice brains were used as internal controls.

### Western Blotting

The protein extracts of mice brains were treated with 6x loading buffer, after mixing and boiling for 10 min, by brief centrifugation to clear the supernatants of the sample. Then SDS-PAGE electrophoresis was performed with concentrated gel concentration of 5% and separation gel concentration of 12% at voltage 80 V for 40 min followed by 120 V until the bromophenol blue runs to the edge of the rubber. Then, the protein bands on the gel were electrotransferred to the nitrocellulose (NC) membrane by a semi-dry method and blocked with 1% bovine serum albumin at 37°C for 1 h followed by washing thrice with 0.05% tween-20 phosphate buffer (PBST). Then the NC membranes were incubated for 2 h with anti-hsp70 rabbit polyclonal antibody and anti-GAPDH rabbit polyclonal antibody and washed 5 times with PBST. Then it was incubated with horseradish peroxidase-labeled Goat anti-Rabbit IgG (H&L) at 37°C for 1 h, followed by five times washing with PBST. The protein bands were analyzed through a chemiluminescence system.

### Cell Viability Assay

Trans Detect® Cell Counting Kit (CCK, TransGen Biotech, Beijing, China) was used for cytotoxicity assay. The viability of RD cells treated with different concentrations of quercetin was determined. Briefly, the RD cells were inoculated into the cell culture plate 1 day in advance, and the medium was discarded the next day, followed by the addition of quercetin at different concentrations. After 48 h of treatment, 10 μL CCK solution was added to each well, cultured for 1–4 h, and the absorbance value was measured at 450 nm.

### Inhibition of Hsp70 by Quercetin and Titration of Virus

RD cells were plated in a 6-well cell culture plate and incubated with different concentrations of quercetin and solvent pretreat cells for 2 h, followed by inoculation of echovirus type 9 with a multiplicity of infection (MOI) = 0.1 cultivated for 48 h, and collected the virus titers in the supernatants. For virus titration experiment, RD cells were placed on 96-well cell culture plates before inoculation. The next day, when the confluence reached about 80%, the supernatant was discarded. Echovirus type 9 was diluted from 10^−1^ to 10^−8^ in a 10-fold series with a cell maintenance fluid, and 6 repeats were performed at each dilution. The virus supernatants of each dilution were inoculated in a monolayer of Vero cells and then cultured in an incubator at 37°C and 5% CO_2_. The occurrence of cytopathic changes was observed and recorded. The echovirus titers in virus culture supernatant and cell supernatant treated with different drug concentrations were calculated by the Reed-Muench method.

### Transfection of Small Interfering RNA (siRNA)

siRNA duplexes targeting genes encoding for human Hsp70 and Control siRNA duplex were first transfected using Lipofectamine RNAiMAX (Thermo Fisher) as per the instructions of the manufacturer, followed by the second transfection after 24 h. Only transfection reagent was treated as normal control. Twenty four hours later, inoculation of echovirus type 9 with a multiplicity of infection (MOI) = 0.1 on RD cells, and after 4 h incubation, the medium was replaced with fresh medium. Cell lysates and culture supernatants were harvested separately at 48 h post-infection. Knockdown of Hsp70 was confirmed by Western blot with antibody against Hsp70, and GAPDH was used as the loading control. The titers of echovirus type 9 culture supernatants were determined by TCID_50_ method as described in section Inhibition of Hsp70 by quercetin and Titration of Virus.

## Results

### Two-Dimensional Gel Electrophoresis Profiles of Echovirus Type 9-Infected Mice Brain

**Figure 1:**
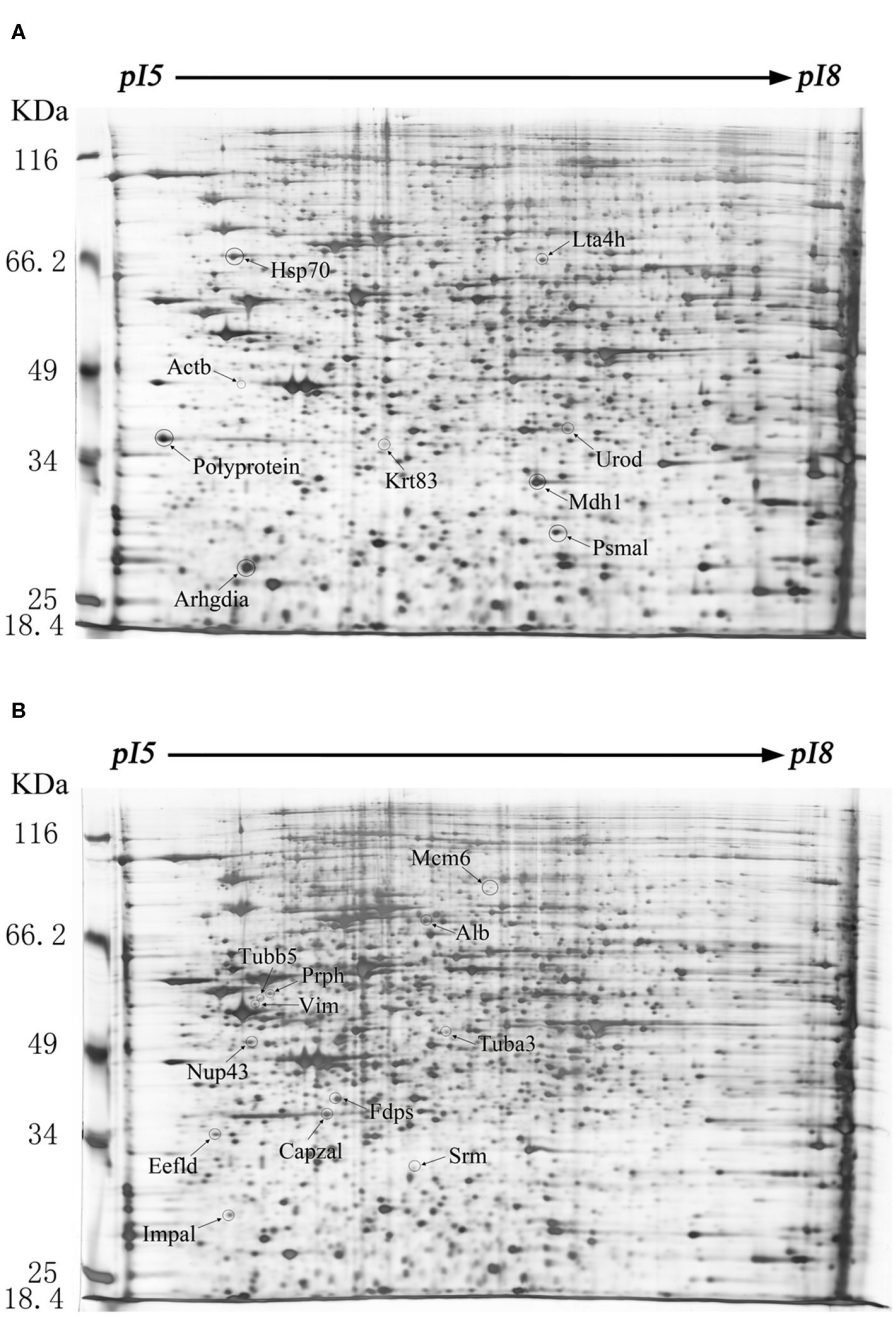
Protein expression profiles of the echovirus type 9-infected and mock-infected mice brains. Equal amounts (250 μg) of brain tissues were separated using the linear gradient IPG strips (24 cm and pI 5-8) and by the 11% SDS-PAGE. Each protein spot on the gel map is assigned a specific number by the software. The up-regulated differentially expressed protein spots were labeled on the gel diagram of the infected tissue samples, while the down-regulated differentially expressed protein spots were labeled on the gel image of the control tissue samples. **(A)** Two-dimensional gel electrophoresis images of echovirus type 9 infected mice brain tissues at 48 h post infection. **(B)** Two-dimensional gel electrophoresis images of mock-infected mice brain tissues at 48 h post treatment.

**Figure 2:**
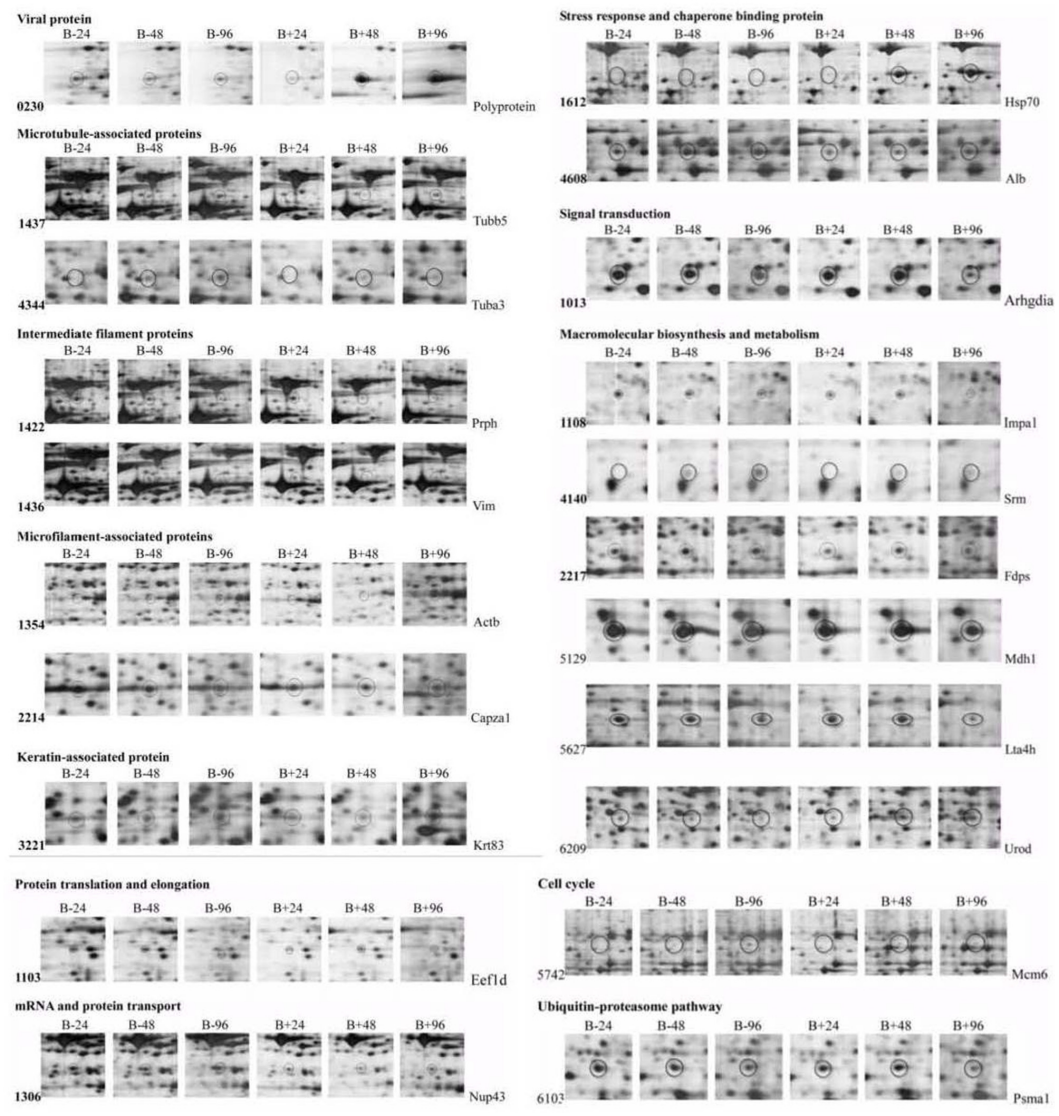
Dynamic profiles of the two dimensional electrophoresis for differentially expressed proteins in the echovirus type 9 infected mice brain tissues. The circles indicate the differentially expressed protein spots in different treatment samples. B+ and B– represent viral infections and control tissue samples, respectively.

The difference of protein expression in the infected and mock-infected mice brains could reflect the response of the target organ of the infected animals. The cellular proteins of echovirus infected and mock-infected mice brains were extracted for 2-DE analysis at 24, 48, and 96 h after the virus challenges. The challenge experiment used six groups of 3 days old mice, 48 mice were randomly divided into mock-infected, and echovirus type 9 infected groups with 8 animals in each group. Proteins were extracted from brain tissue samples treated at different times and compared by two-dimensional electrophoresis, with four replicates for each sample. In the gel analysis of two-dimensional electrophoresis, the detected protein ranged from 1,700 to 2,000 spots. In total, 20 host proteins showed altered expression levels in the echovirus type 9 infected mice brains compared with the mock-infected mice brains. Of these, nine were significantly up-regulated (ratio of infection/mock ≥2, *p* ≤ 0.05, Figure 1A), while the remaining twelve were down-regulated (ratio of infection/mock ≤ 0.5, *p* ≤ 0.05, Figure 1B). Of the nine up-regulated proteins in echovirus infected mice brains, one protein spot induced at 24 h post-infection was identified to be new that showed increased expression levels with the infection time, while one protein spot first disappeared at 24 h post-infection, down-regulated at 48 h post-infection, was up-regulated significantly at 96 h post-infection (Figure 2, Table 1). Among the 11 down-regulated protein spots, five protein spots were completely inhibited at 24 h post-infection, two protein spots were completely inhibited at 48 h post-infection, and one protein spot was disappeared at 96 h post-infection. These suggest that viral infection does cause changes in the expression profile of brain tissue in the host, which may provide useful clues to elucidate its pathogenesis.

**Table 1:** Basic information for the differentially expressed proteins that were successfully identified by Mass spectrometry.

**Spot no.^(a)^**	**Protein name**	**Abbr**.	**Accession No.^(b)^**	**Mr (pred/exp)^(c)^**	**p*I* (pred/exp)**	**Matched/unmatched^(d)^**	**Abundance Ratio_infected/uninfected_ (Means±SD)^(e)^**			**Coverage(%)^(f)^**	**Protein/best ion score^(g)^**	**Peptides Identified^(h)^**
							**24 h**	**48 h**	**96 h**			
**Viral protein of echovirus**												
0230	Polyprotein	Polypro	gi|769800	245.09/39.5	7.1/5.2	18/64	0.46 ± 0.03	8.18 ± 0.16*	10.63 ± 0.13	40	254/101	NAGSINAPTVSDSRA
**Cytoskeleton proteins**												
**Microtubule-associated proteins**												
1437	tubulin, beta 5	Tubb5	gi|7106439	49.64/55.30	4.78/5.42	14/34	ND^(k)^	ND	0.75 ± 0.24*	63	181/61	GHYTEGAELVDSVLDVVR
4344	tubulin alpha-3 chain	Tuba3	gi|88683157	32.92/51.12	5.86/6.3	8/121	ND	0.66 ± 0.33**	0.31 ± 0.06***	56	121/18	LIGQIVSSITASLR
**Intermediate filament proteins**												
1422	Peripherin	Prph1	gi|2253159	52.65/56.5	5.36/5.5	15/27	0.74 ± 0.05*	0.43 ± 0.07**^(j)^	0.66 ± 0.13*	57	197/74	FLEQQNAALR
1436	Vimentin	Vim	gi|2078001	51.53/56.1	4.96/5.6	21/30	0.80 ± 0.20	ND	ND	58	289/41	ISLPLPTFSSLNLR
**Microfilament-associated proteins**												
1354	put. beta-actin (aa 27-375)	Actb	gi|49868	39.16/40.1	5.78/5.82	9/37	ND	0.52 ± 0.01**	2.24 ± 0.27**	25	168/71	SYELPDGQVITIGNER
2214	F-actin-capping protein subunit alpha-1	Capza1	gi|34223730	32.92/40.10	5.34/5.85	6/122	0.30 ± 0.15**	0.48 ± 0.00**	0.64 ± 0.18	13	128/44	LLLNNDNLLR
**Keratin-associated proteins**												
3221	Keratin 83	Krt83	gi|134085840	53.95/41.3	5.43/6.1	14/27	0.64 ± 0.74	0.91 ± 0.08	2.32 ± 0.50**	18	105/0	LLEGEEQR
**Signal transduction**												
1013	Rho GDP dissociation inhibitor (GDI) alpha	Arhgdia	gi|31982030	23.39/27.31	5.12/5.21	9/269	1.27 ± 0.12	1.15 ± 0.15	0.43 ± 0.20**	76	278/51	SIQEIQELDKDDESLR
**mRNA and protein transport**												
1306	Nucleoporin 43	Nup43	gi|166295220	43.73/51.6	5.09/5.5	5/103	0.59 ± 0.14*	0.41 ± 0.05**	0.64 ± 0.13*	32	108/37	TIDNADSSTLHAVTFLR
**Cell cycle**												
5742	Minichromosome maintenance complex component 6	Mcm6	gi|6678832	92.81/95.31	5.32/6.63	8/77	ND	0.87 ± 0.31	0.39 ± 0.17**	37	96/25	DFYVAFQDLPTR
**Stress response/chaperone binding proteins**												
1612	Heat shock protein hsp70	Hsp70	gi|445605	75.47/75.30	5.22/5.3	7/91	NI^(i)^	NI	NI	36	98/66	DIDEVILVGGSTR
4608	Serum albumin	Alb	gi|1351907	69.25/80.00	5.82/6.2	7/54	0.65 ± 0.19	0.35 ± 0.07**	0.33 ± 0.04**	34	152/40	DAFLGSFLYEYSR
**Ubiquitin-proteasome pathway**												
6103	Proteasome (prosome, macropain) subunit, alpha type 1	Psma1	gi|33563282	29.53/30.1	6 /7.0	12/84	0.97 ± 0.30	1.32 ± 0.19*	0.46 ± 0.12**	65	204/62	NQYDNDVTVWSPQGR
**Protein translation and elongation**												
1103	Elongation factor 1-delta	Eef1d	gi|13124192	31.27/35.8	4.91/5.24	8/43	0.39 ± 0.03**	0.74 ± 0.11	0.56 ± 0.06*	53	162/27	FYEQMNGPVTSGSR
**Macromolecular biosynthesis and metabolism**												
1108	Inositol monophosphatase	Impa1	gi|3914098	30.42/29.7	5.08/5.31	9/63	0.75 ± 0.01*	0.85 ± 0.01	0.32 ± 0.08**	44	170/32	EIEIIPLQR
4140	Spermidine synthase	Srm	gi|6678131	34.04/32.81	5.4/5.9	7/93	ND	0.55 ± 0.06**	0.46 ± 0.00**	30	167/30	AAFVLPEFTR VLIIGGGDGGVLR
2217	Farnesyl diphosphate synthetase	Fdps	gi|56789674	40.54/40.32	5.49/5.95	7/90	0.81 ± 0.00*	0.63 ± 0.12*	0.44 ± 0.04**	63	161/61	ELGHPEIGDAIAR
5129	Malate dehydrogenase 1,NAD (soluble)	Mdh1	gi|254540027	36.49/33.64	6.16/6.84	6/126	1.34 ± 0.07**	1.46 ± 0.06**	0.42 ± 0.08**	44	173/58	FVEGLPINDFSR
5627	Leukotriene A-4 hydrolase	Lta4h	gi|198884	68.96/68.50	5.91/6.78	7/35	1.28 ± 0.02**	1.05 ± 0.27	0.29 ± 0.02**	52	117/42	TLTGTAALTVQSQEENLR
6209	Uroporphyrinogen decarboxylase	Urod	gi|148698629	40.66/41.10	6.21/6.9	8/54	1.05 ± 0.12	4.50 ± 0.30***	2.85 ± 0.05***	62	144/63	FALPYIR

(a) *Spot no. is the unique sample spot protein number that refers to the labels in Figure 1*.

(b) *Accession no. is the MASCOT result of MALDI-TOF/TOF searched from the NCBInr database*.

(c) *pred/exp, predicted/experimental*.

(d) *The number of peaks that match/do not match the trypsin peptides*.

(e) *Mean, the average abundance ratio for paired protein samples. SD means the standard deviation of abundance ratios of the one certain protein spot. The protein spots with abundance ratio infected/uninfected <0.5 were down regulated, and >2 were up regulated*.

(f) *Sequence coverage (%) is the number of amino acids spanned by the assigned peptides divided by the sequence length*.

(g) *Protein score (based on combined MS and MS/MS spectra) and best ion score (based on MS/MS spectra) were from MALDI-TOF/TOF identification. The proteins that had a statistically significant protein score of >82 (p ≤ 0.05) were considered successfully identified*.

(h) *The peptides identified by MALDI-TOF/TOF with statistically significant ion score (confidence interval, ≥95%) or ion score above 21*.

(i) *NI, the protein spots were newly induced in Echovirus-infected tissues*.

(j) The p-values of paired T-test. *p ≤ 0.05, **p ≤ 0.01, ***p ≤ 0.001.

(k) *ND, the protein spots were not detectable in Echovirus-infected tissues*.

### Identification of the Differentially Expressed Proteins by MS/MS Mass Spectrometry

**Figure 3:**
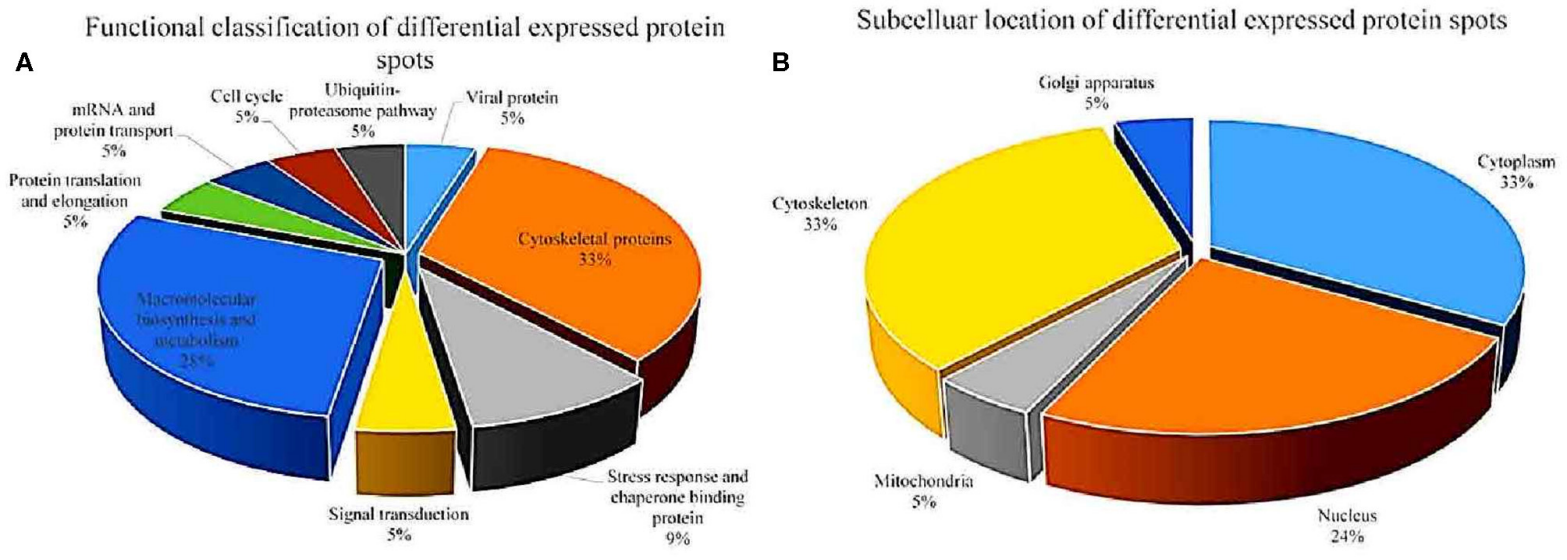
Functional classfication **(A)** and subcellular distribution **(B)** of the differentially expressed proteins in echovirus infected mice brain tissues.

After analysis by PDQuest software, 21 protein spots with significant differences were extracted from two-dimensional electrophoresis gel and subsequently identified by mass spectrometry. The annotation of the differentially expressed proteins using the UniProt Knowledgebase (Swiss-Prot/TrEMBL) and Gene Ontology database identified their functions (Table 1, Figure 3). The 21 differentially expressed protein spots were found to correspond to the following proteins: one viral protein (5%), seven cytoskeletal proteins (33%), six macromolecular biosynthesis and metabolism (28%), two stress response and chaperone binding proteins (9%), five other cellular proteins (25%). These differentially expressed proteins were located mainly in the cytoplasm (33%), cytoskeleton (33%), and nucleus (24%), also distributed within the mitochondria (5%) and Golgi apparatus (5%).

### Validation of the Differentially Expressed Proteins in Mice Brain Tissues During Echovirus Infection by Quantitative Real-Time RT-PCR

**Figure 4:**
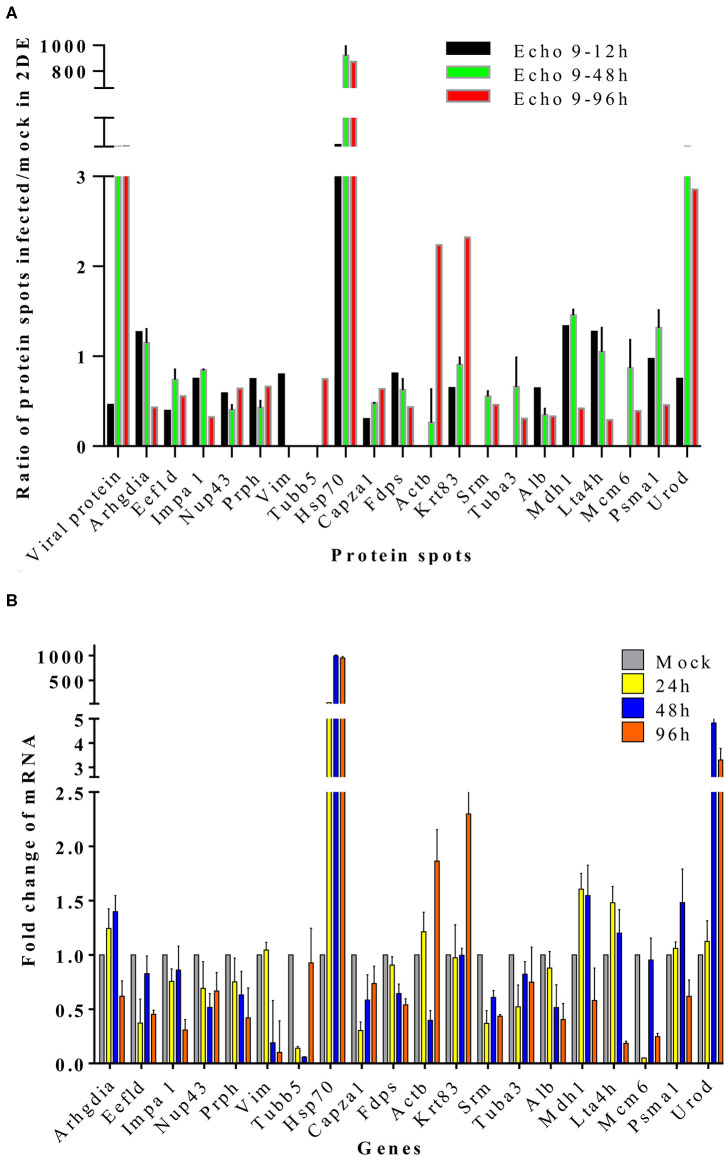
Translational and transcriptional alterations of differentially expressed proteins in echovirus-infected mice brain tissues. **(A)** Quantification of the differentially expressed proteins at translational levels, the densities of the spots (total pixel intensity within spot boundaries) in two dimentional electrophoresis gel maps were analyzed by PDQuest 8.0.1 software. **(B)** Quantification of the differentially expressed proteins in mice brains tissuses at transcriptional levels by qPCR. Transcription of the genes in the samples were normalized to the internal control GAPDH housekeeping gene, and the mock-infected mice brain tissue samples at each time point were set as the reference. The values were the ratios of the increase or decrease in gene transcription in the infected sample relative to the control sample, and mock-infected tissues samples were normalized to 1. Error bars represent standard deviations.

To further validate the proteins identified by 2DE and MS/MS method for the echovirus infected and mock-infected samples, the transcriptional levels of the differential expressed proteins were analyzed by quantitative RT-PCR method, and the GAPDH gene was used as the internal control. More than 2-fold (ΔΔCT≥1, *p* ≤ 0.05) changes in relative expression level were considered significant. In general, the protein expression profiles (Figure 4A) and the results of quantitative real-time RT-PCR in the detection of gene transcripts (Figure 4B) were found to be mostly consistent, with only minor inconsistency. Of these genes, the abundance of transcripts of genes Prph, Vim, Fdps, and Alb were continuously downregulated from 24 to 96 h after infection, while the transcripts of genes Arhgdia, Actb, Krt83, Mdh1, Mdh1, Lta4h, and Psma1 were up-regulated at different times after infection, and some of them also showed a wavy up-down expression pattern. It is worth noting that with the extension of virus infection time, the transcript abundance of Hsp70 and Urod continued to increase, which was completely consistent with the protein expression trend on a two-dimensional electrophoresis gel. Since the process of gene transcription to expression involves post-transcriptional modification and post-translational modification, it is very common that the results of qPCR are not completely consistent with the protein expression, but it can confirm the detection results of the protein to some extent. These transcription profile data provided a useful complement to the differential protein profile detected by proteomics analysis.

### STRING Analysis of the Relationships Between the Differentially Expressed Proteins of Mice Brain

**Figure 5:**
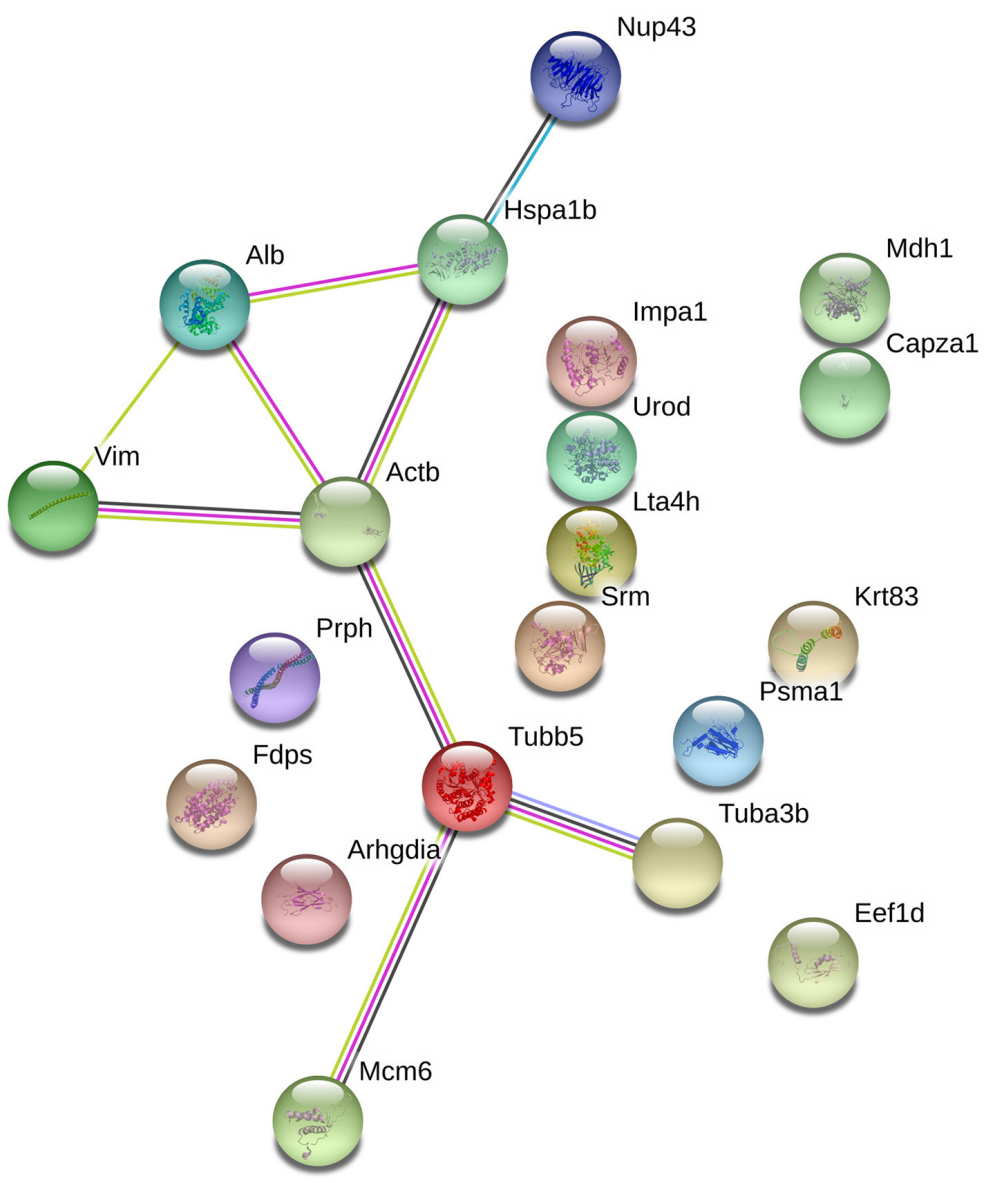
The protein-protein interaction network of the differentially epxressed proteins that induced by echovirus infection and analyzed by STRING software.

With the goal of exploring the details of the potential network relationships between differentially regulated proteins, the STRING tool version 11.0 was used. The differentially expressed proteins identified in this study mainly formed one functional network (Figure 5). The specific network had at least three “focus” proteins (Hspa1b, Actb, and Tubb5). The network of these proteins corresponded to a wide variety of cellular processes, including protection of the proteome from stress, folding and transport of newly synthesized polypeptides, various types of cell motility, maintains cell shape, movement, and intracellular transport.

### Western Blot Confirmation of the Expression of Hsp70

**Figure 6:**
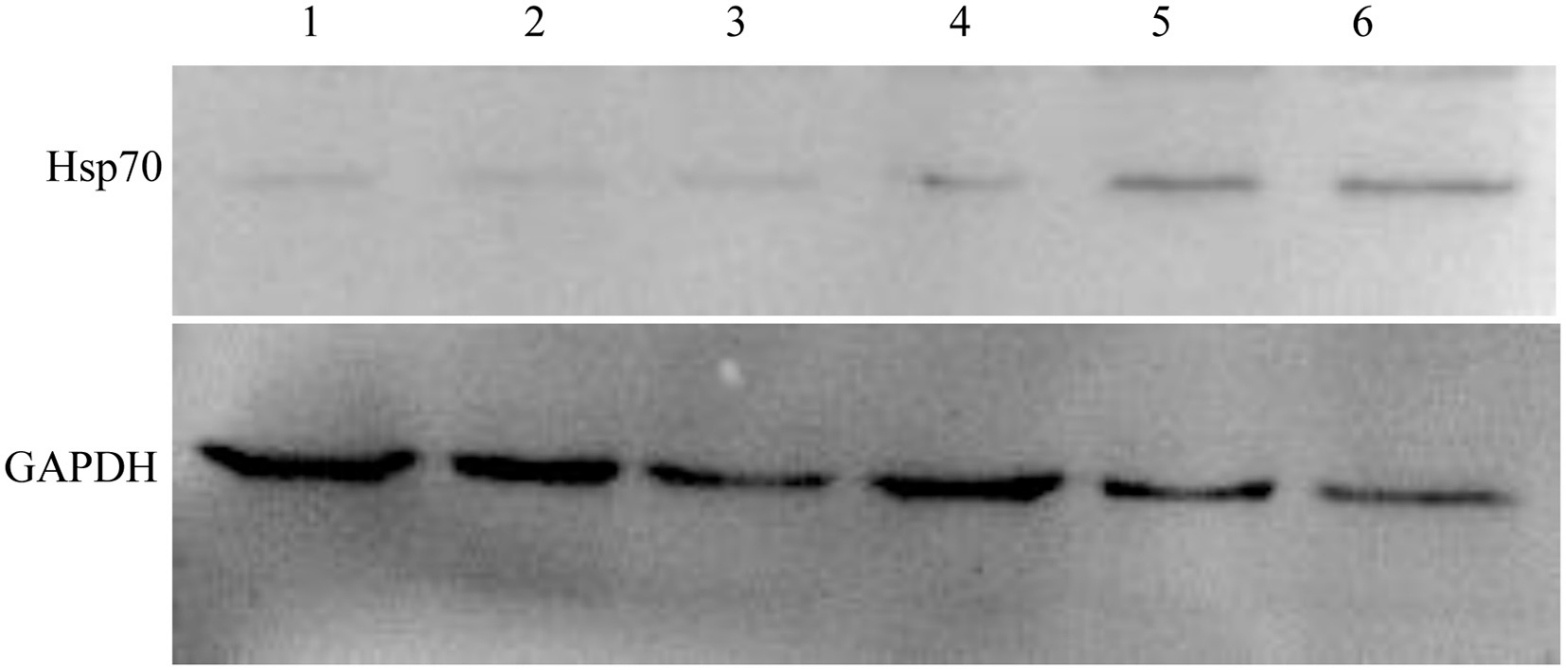
Western blot confirmation of representative proteins in echovirus-infected mice brain tissues. The immunoblots of Hsp70 and GAPDH with antibodies against Hsp70 and GAPDH, respectively.

We chose the Hsp70, whose variation trend was consistent on fluorescence quantitative PCR and two-dimensional electrophoresis gel, to further validate its expression by Western blot analysis. Figure 6 shows that Hsp70 was up-regulated in echovirus infected mice brain since 24 h post-infection. These results were also consistent with those obtained from both the MS/MS and quantitative RT-PCR.

### Inhition of Hsp70 Results in Reduction of Infectious Echovirus Type 9 Progeny

**Figure 7:**
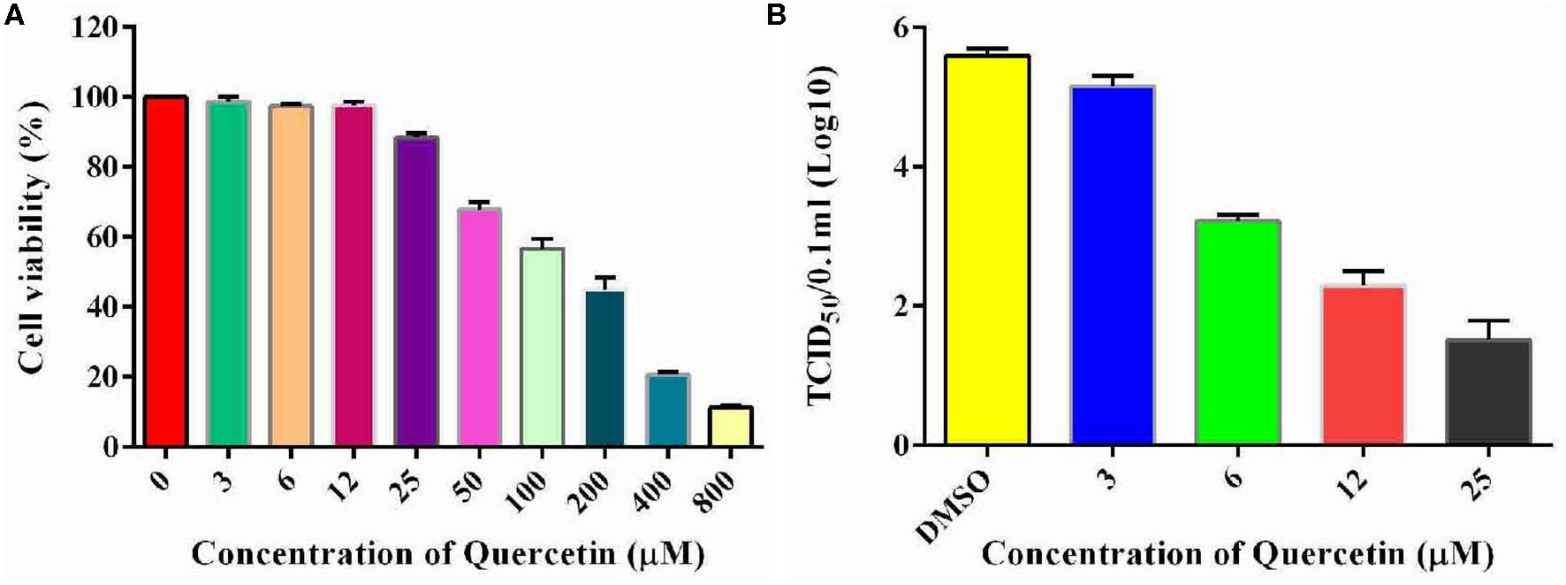
Inhibitory potential of quercetin on the replication of echovirus in RD cells. **(A)** Effects of different concentrations of Hsp70 inhibitor quercetin on RD cell viability detected by CCK assay. The absorbence of the cells treated with DMSO were normalized to 100%. **(B)** Echovirus titers detected in the presence and absence of different concentrations of Hsp70 inhibitor quercetin 2 days post-infection.

To examine the potential role of Hsp70 in the life cycle of echovirus type 9, we further performed the *in vitro* inhibition experiment of Hsp70 by its inhibitor quercetin on RD cells. To exclude the possibility that the cytotoxicity affects the replication of echovirus, the viability of cells treated with different concentrations of quercetin were detected by CCK assay, and the optimum working concentration of the drug was determined. The results showed that the viability of quercetin treated cells decreased slightly in comparison with the solvent treated control group (*p* > 0.05, Figure 7A) at a concentration of 25 μM. Subsequently, echovirus type 9 was inoculated on the RD cells with the multiplicity of infection of 0.1 in the presence of different concentrations of quercetin, and replication of echovirus was determined by titer determination (TCID_50_). The data showed that the virus titers were significantly decreased (*p* < 0.05, Figure 7B) even at the quercetin concentration of 3 μM, and has the obvious concentration gradient dependence effect.

### Hsp70 Is Required for the Infection of Echovirus Type 9

**Figure 8:**
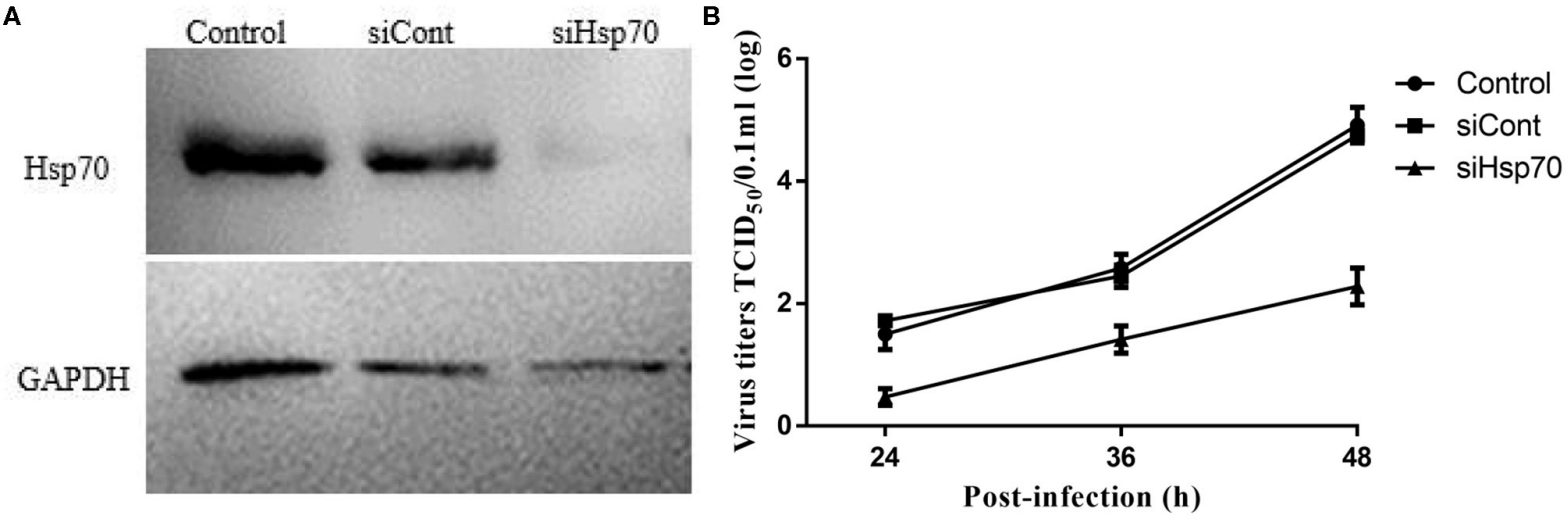
Knockdown of Hsp70 in echovirus type 9-infected cells. **(A)** The efficiency of siRNA knockdown in siRNA duplexes transfected RD cells, the expression of Hsp70 was confirmed by Western blot. **(B)** The echovirus titers in the RD cells supernatants were measured by TCID50 test.

Given that quercetin can inhibit the production of the progeny of echovirus type 9, and in addition to Hsp70, quercetin may also inhibit many enzyme systems including tyrosine protein kinase, phospholipase A2, phosphodiesterases, mitochondrial ATPase, PI 3-kinase and protein kinase C, we wondered whether quercetin's inhibition of echovirus was achieved by its primary target, Hsp70. As shown in Figure 8A, compared with control group of scrambe siRNA and transfect reagents, silencing of Hsp70 expression by siRNAs had significantly reduced the expression of Hsp 70. And a significant inhibition of echovirus titers was observed in culture supernatants of Hsp70 knockdown RD cells (Figure 8B). Thus, our results further indicated that quercetin inhibited echovirus replication by Hsp70.

## Discussion

Echovirus type 9 is one of the major culprits of viral pneumonia and encephalitis in neonates, infants, and young children. The harmful effects of echovirus type 9 are obvious, but its pathogenesis is little studied. A lot of evidence emphasizing the possibility that comparative proteomics for the screening of the differentially expressed proteins, which associate with the physiological and pathological process of viral infection at cellular levels, could explore the underlying mechanism. In this study, we obtained a dynamic overview of the profile of the differentially expressed proteins in the mice brains responding to echovirus type 9 infections. The identified host proteins were shown to be involved in cytoskeleton organization, mRNA and protein transport, protein translation and elongation, signal transduction, stress response, and chaperone-mediated protein folding, ubiquitin-proteasome proteolytic system, and macromolecular biosynthesis and cell cycle regulation. Therefore, it is a multifaceted regulatory network.

### Effects of Echovirus Type 9 Infection on Proteins Associated With Cytoskeleton

Cytoskeleton associated proteins usually are ubiquitous cellular proteins that form the basis for the structure and integrity of cells. While viruses are obligate intracellular parasites that have a life cycle requiring the engagements and modification of cytoskeleton at all stages, from invasion through replication to budding and transmission. By far, lots of viruses have been reported that evolved to engage and subvert the cytoskeleton proteins' function, such as RSV (Shahriari et al., 2016), Dengue Virus (Wang et al., 2010), Rubella virus (Krater et al., 2018), avian influenza viruses (Guo et al., 2018), rabies virus (Monroy-Gomez et al., 2018), Rhinovirus (Michi and Proud, 2018). In the present study, the microtubule-associated proteins Tubb5, Tuba3, the intermediate filament proteins Prph, Vim, and microfilament-associated protein Capza1 were down-regulated by echovirus infection, while microfilament-associated protein Actb and keratin-associated protein Krt83 were up-regulated. The downregulation of the cytoskeleton protein indicated the possible involvement of the virus in the disruption of the cytoskeleton, a critical step for the viral particles' release. The decrease of F-actin-capping protein subunit alpha-1 (Capza1) have been detected in several viruses infected cells, such as Avian Influenza Virus (Ding et al., 2016; Li et al., 2017), infectious bursal disease virus (Wu et al., 2012), and even directly interact with viral protein (Mutso et al., 2018), may be related to the death of cells infected with virus resulting the cytopathic effects.

### Proteins Involved in Stress

The life cycle of viruses is intimately tied to the life cycle of the host cells and the capability of viruses to evolve specific mechanisms to block their host's immune response and use their host function to service their replication has been well-documented. Heat shock proteins belong to the group of molecular chaperones, and Hsp70 is one of the key members. Albumin (Alb) also is a major plasma protein target of oxidant stress (Himmelfarb and Mcmonagle, 2001). Of the 21 differentially expressed proteins detected, both Hsp70 and Alb involved in cellular stress responses were strongly induced after echovirus type 9 infections representing a significant finding in the present study. Several studies have reported the up-regulation (Lim et al., 2005; Lahaye et al., 2012) as well as downregulation (Wu et al., 2012) of Hsp70 in virus infection. Hsp70 is required at different steps of the life cycle of the virus, such as viral entry, RNA replication, and virion biogenesis (Taguwa et al., 2015). Moreover, Hsp70 functions directly by interacting with viral proteins and also by interacting directly with viral nucleic acids (Nanda et al., 2004; Oh and Song, 2006; Couturier et al., 2010; Lahaye et al., 2012). Preliminary functional verification results in this study indicated that Hsp70 is a host factor hijacked by echovirus, and its up-regulated expression promotes viral proliferation. However, the exact mechanism of its action remains to be further studied.

### Proteins Involved in Macromolecular Biosynthesis and Metabolism

In this study, except Mdh1 and Urod, most of the macromolecular biosynthesis and metabolism-related proteins, Impa1, Srm, Fdps, and Lta4h, were all down-regulated, this is coincident with a previous report (Wu et al., 2012). The fifth enzyme of the heme biosynthetic pathway, uroporphyrinogen decarboxylase (Urod), is a housekeeping enzyme, whose activity is enhanced during erythropoietic differentiation (Romana et al., 1987). The deficiency of this gene usually resulted in the abnormal porphyrin metabolism and associated with porphyria cutanea tarda (Nakano et al., 2018), and related research reports are relatively few. Impa1 (Inositol monophosphatase 1), is responsible for the provision of inositol required for the synthesis of phosphatidylinositol and polyphosphoinositides, and also has been implicated as the pharmacological target for lithium action in the brain. It is a putative target for lithium in the treatment of bipolar disorder (Agam et al., 2009). By far, IMPase (IMPA1) is the gene known to encode IMPase activity, which can be inhibited by lithium with a therapeutic concentration (~1 mM) (De Meyer et al., 2011). It might be worth a try in the future to see whether it works against echovirus by lithium using the experiments of *in vitro* and *in vivo*.

### Proteins Involved in UPP

A large number of viral members, including DNA and RNA viruses, have evolved the ability to regulate the ubiquitin-proteasome system for different reasons, such as immune escape, viral invasion or release, transcriptional regulation, and apoptosis suppression (Gilfoy et al., 2009). The eukaryotic 26S proteasome is a ubiquitous multi-protease complex, which was composed by a barrel-shaped, proteolytic core complex, and highly conserved. As the main protein quality control of eukaryotic cells, proteasome controls the assembly, connection, function, and synaptic signal of neurons (Pak and Sheng, 2003). One of the differentially expressed proteins, namely proteasomal subunit α-1 (Psma1), was identified to be down-regulated in this study. Psma1, as one subunit of the 26S proteasome, was reported to bind the HBV regulatory X protein (HBx) through the C-terminal region of the transactivation domain in a competitive manner (Zhang et al., 2000). Mitochondrial dysfunction, oxidative modification of the proteasome subunit, and changes in the expression level of the proteasome subunit all affect the activity of the proteasome, leading to neurological dysfunction. Down-regulation of Psma1 on human brain tissue infected by street rabies virus has been reported previously (Farahtaj et al., 2013). According to our data, decreased levels of Psma1 in echovirus infected sample group may be an evidence of proteasomal dysfunctional in echovirus type 9 infected mice brain tissues.

### Possibility of Host Protein Hsp70 as the Therapeutic Target of Echovirus

In a large number of previous studies, people used to screen a large number of compounds for the therapeutic activity of specific diseases, and then study the mechanism of drug action after screening specific drugs. By using proteomics, potential drug targets can be directly found. If there are available protein inhibitors, antagonists or marketed drugs, the therapeutic effect can be directly evaluated, which can greatly shorten the process of drug development, and the mechanism is easy to clarify. Based on the finding of Hsp70 in the differentially expressed proteins in echovirus type 9 infected mouse brain, the role of Hsp70 was further established by siRNA mediating silencing of Hsp70 and quercetin which significantly limited the infection of echovirus type 9 in RD cells. Given the low level of Hsp70 expression in normal cells, and Hsp70 controls diverse cellular functions of the body, this provides good opportunities for the screening of chemical compounds that inhibit disease-related Hsp70 activities. There is an increasing interest in Hsp70 as potential drug targets for the treatment of various diseases, especially for cancers (Jego et al., 2013) and virus diseases (Lahaye et al., 2012; Gao et al., 2014; Pujhari et al., 2019). Hsp70 is thought to play a role in the stabilization of cellular proteins required for normal protein folding and stabilization. The involvement of these physiological activities requires ATPase activity, and like many heat shock proteins, Hsp70 has an ATP-binding domain. This characteristic makes these proteins highly druggable, and the development of specific inhibitors has attracted wide attention in recent years. Our immediate goal is to find the potential targets for echovirus infection disease through the construction of virus infection model, and test potency of Hsp70. The greatest highlight of our study is the possibility of Hsp70 as a successful therapeutic target for echovirus infection, which was confirmed by a variety of different technical data. As for the clinical therapeutic effect of quercetin and the mechanism of Hsp70 in echovirus infection, further study is still needed.

## Conclusions

In this study, a comparative proteomic analysis was carried out to investigate the differential expression profile of the brain between echovirus type 9 infected and mock-infected mice. The analysis of the proteomic study identified 21 differential expressed host proteins and found that 9 proteins were upregulated and 11 downregulated in echovirus type 9 infected mice brain. The identified differentially expressed host proteins mainly belong to macromolecular biosynthesis and metabolism, cytoskeleton, stress response, and chaperone binding proteins in function, while their subcellular distribution was mainly in the cytoplasm, cytoskeleton, and nucleus. One of the differential host proteins, Hsp70, was chosen to validate the reliability of the proteomic data, and the Western blotting result was consistent with the proteomic data. We further evaluated the function of Hsp70 using quercetin, an inhibitor of Hsp70, and Hsp70 targeted siRNAs, the results showed that the differentially expressed protein Hsp70 was necessary for the infection of echovirus type 9 in the brain. Moreover, we also speculated that the differential proteins found in this study could have important potential application value, which could used in the therapy of viral pneumonia caused by echovirus type 9; however, that needs to be validated.

## Data Availability Statement

The raw data supporting the conclusions of this article will be made available by the authors, without undue reservation, to any qualified researcher.

## Ethics Statement

The study was approved in 2017 by the First Affiliated Hospital of Zhengzhou University (Study approval number: 2017050).

## Author Contributions

YW and DM: conceptualization. QW and HL: methodology. DW, HZ, and XD: validation. FL: formal analysis. YW: writing–original draft preparation and funding acquisition. FL, QW, JW, and DM: writing–review and editing. HZ: visualization. HL: supervision. JW: project administration. All authors contributed to the article and approved the submitted version.

## Conflict of Interest

The authors declare that the research was conducted in the absence of any commercial or financial relationships that could be construed as a potential conflict of interest.
