# Supplementary material for: Hsp70 Is a Potential Therapeutic Target for Echovirus 9 Infection
Source: Front Mol Biosci. 2020 Jul 17;7:146. doi: 10.3389/fmolb.2020.00146 (PMC7379509; doi:10.3389/fmolb.2020.00146)
Supplement: Supplementary file 1 [file Table_1.doc]

| Gene | Forward primer （5’→ 3’） | Reverse primer （5’→ 3’） | Accession No |
| --- | --- | --- | --- |
| GAPDH | GGAGAAACCTGCCAAGTATGA | CCTGTTGCTGTAGCCGTATT | NM_001289726.1 |
| Arhgdia | ACTGGACAAGGACGATGAAAG | CTGTTCACCCGGAAAGAGATT | NC_000077.6 |
| Eef1d | GCCAGAGAGAACATCCAGAAA | CACTTGACGCATAGGAGAGAC | NM_001285429.1 |
| Impa 1 | TGTACAGCTGTGTGGAAGATAAG | GCAGTGGATTCCCATCTCATAA | NM_018864.6 |
| Nup43 | ATTCTGACGGAGGGTTTGAAG | GCTGGGACCTGTATGGTAATG | NM_145706.2 |
| Actb | GACGGCCAAGTCATCACTATT | TAGGAGCCAGAGCAGTAATCT | X03765.1 |
| Prph | CCTCAAGAAGCTACACGAAGAG | GCTCTCGCACTTTCGACTTAT | XM_006520631.4 |
| Vim | CTCGTCACCTTCGTGAATACC | CTCTGGTCTCAACCGTCTTAATC | M26251.1 |
| Tubb5 | GCTAAGTTCTGGGAGGTGATAAG | CGAAGTTGTCTGGTCTGAAGAT | NM_011655.5 |
| Hsp70 | TAAACGTGCTGCGGATCAT | GTCCTTCTTGTGCTTCCTCTT | NM_010478.2 |
| Capza1 | GATGAAGAGAAGGTCCGCATAG | CAGTACAGAAGCCGTTGGAATA | NM_009797.3 |
| Fdps | GGTATCAGAAGCCAGGCATAG | CCGCAATAGGCAGGTAGAAA | CT010365.1 |
| Krt83 | GAGGGCTACAAGAAGAGGTATG | TGTGTCTGAGATGTGGGAATG | NM_001201323.1 |
| Srm | GCCTCTTCAAGGAGTCCTATTAC | AAGTTGGTGCTCGGGTTT | L19311.1 |
| Tuba3 | AGGGAGTGTCTCTCCATTCA | CCACACGGATCCCATCTATAAC | NM_001033879.3 |
| Alb | CTAGTTCGCTACACCCAGAAAG | GCAGATATCAGAGTGGAAGGTG | NM_009654.4 |
| Mdh1 | GGCACAGCCTTGGAGAAATA | CTGGATACTGGGTCGATGAATG | NM_008618.3 |
| Lta4h | GAGAAAGAGCAGGTGGAGAAAT | AAGTGATCCCACGTCTTGTTAG | NM_008517.2 |
| Mcm6 | TCGAGCCTTGAAGACCTTTG | CTGCTGCTCTACGTCCTTAATC | NM_008567.2 |
| Psma1 | GGTATCTCAATTGCGGGTCTAA | GAGGGCCCATATCATCATAACC | NM_011965.2 |
| Urod | GGCTATGAGGTAGTTGGACTTG | GAAGCAGGCGTGAATGTTTG | XM_006502931.2 |

**Table S1** The primers for Real-time RT-PCR
